# Genome Wide Association Studies: Identifying the Genes that Determine the Risk of Abdominal Aortic Aneurysm

**DOI:** 10.1016/j.ejvs.2008.06.003

**Published:** 2008-10

**Authors:** The Aneurysm Consortium

**Affiliations:** c/o Robert Kilpatrick Clinical Sciences Building, Leicester Royal Infirmary, Leicester LE2 7LX, UK; m.bown@le.ac.uk

Abdominal aortic aneurysm (AAA) is a multifactorial disease with a strong genetic component. Aside from the major environmental risk factor of tobacco use, and the un-modifiable risk factors of advancing age and male gender, there is compelling evidence for the genetic contribution to the pathogenesis of AAA, with up to ten-fold increased risk in first degree relatives of those with AAA.^1^ However, it is widely accepted that AAA is unlikely to be associated with a single gene, and that multiple genetic factors are responsible.^2^ This makes identifying the genes involved in AAA challenging, as conventional approaches that have been successful for monogenic diseases such as cystic fibrosis and Huntingtons disease are not appropriate.

One approach has been through hypothesis-driven association studies, whereby the frequency of a common variant in a candidate gene is compared between a group of unrelated affected individuals and unaffected age-matched controls. The major limitation of this approach is that the selection of appropriate candidate genes relies on knowledge of the pathological processes underlying the disease in question, and since this is currently unclear for AAA, the choice of genes studied to date has been made on a ‘best guess’ basis. These studies have recently been reviewed^3^ and the genes that have been studied include inflammatory mediators, tissue proteases and their inhibitors, those involved in endothelial and smooth muscle cell biology and those coding for components of the renin-angiotensin system. While such approaches have often given inconsistent results in different laboratories, meta-analysis of the available data has shown some evidence for modest risk effects associated with common variants in the genes for Angiotensin Converting Enzyme (Odds Ratio (OR) 1.33, Methylenetetrahydrofolate reductase (OR 1.14)), and matrix metalloprotease-9 (OR 1.09).

A complementary approach has recently become available due to advances in genomic investigation and bioinformatics that have allowed significant proportions of the common variation in the human genome to be identified and recorded. These variations consist mainly of single nucleotide polymorphisms (SNPs) (DNA nucleotide substitutions) and to date more than 6 million have been identified and verified.^4^

Recent technological advances have enabled us to examine this variation in an affordable way. Array-based platforms now exist that allow over a million SNPs to be assayed in a single experiment. This allows us to carry out Genome-Wide Association Studies (GWAS), and to compare the variation seen in those with a particular disease to groups of healthy controls and thus to identify the genes associated with the disease in a hypothesis-free approach.

However GWAS have particular design and analysis issues,^5^ principally sample size requirements. Using conventional statistical analyses with alpha = 0.05 if 500 000 SNPs were assayed in the same experiment 100 000 significant results would be expected, so clearly adjustment for multiple hypothesis testing must be made. Different methods exist to allow for this, including the well-known Bonferroni correction which in this example requires us to generate a P-value of less than 1 × 10^−7^ to classify a result as significant. Because of this, sample sizes of at least 2000 cases and 2000 controls are required. Any SNPs showing a statistically significant effect will then need to be genotyped in a second (replication) cohort of similar size to the initial study cohort and so overall large numbers of both cases and controls are required.

The utility of GWAS as a tool for genetic investigation of complex disease has recently been demonstrated by the Wellcome Trust Case-Control Consortium^6^ with many new loci being identified for heart disease, type 2 diabetes etc. (as well as confirming the contribution of several “candidate” genes). For AAA this approach has already borne fruit, with a SNP on chromosome 9p21 that was originally identified as being associated with heart disease recently reported to also be associated with AAA (OR = 1.3).^7^ The nearest genes to this SNP are a cluster consisting of *CDKN2A-ARF-CDKN2B*, which code for proteins that play a role in cell-proliferation, senescence and apoptosis, all features implicated in atherogenesis. The potential mechanism, by which variants in this chromosome 9 region increase risk of CHD or AAA, remains to be elucidated.

To exploit the new molecular technologies, an international group of investigators from Australia, New Zealand and the UK have formed “The Aneurysm Consortium” and recently obtained funding to carry out a GWAS of AAA. Hopefully this will provide insights into the pathogenesis of the disease and may identify novel potential targets for pharmacotherapeutic intervention for small AAA.

The GWA approach also has the potential to drive forward knowledge of the aetiologies of venous disease and other forms of peripheral atherosclerosis.

## References

[1] Johansen K., Koepsell T. (1986). Familial tendency for abdominal aortic aneurysms. JAMA.

[2] Kuivaniemi H., Shibamura H., Arthur C., Berguer R., Cole C.W., Juvonen T. (2003). Familial abdominal aortic aneurysms: collection of 233 multiplex families. Journal of Vascular Surgery.

[3] Thompson A.R., Drenos F., Hafez H., Humphries S.E. (2008). Candidate gene association studies in abdominal aortic aneurysm disease: a review and meta-analysis. European Journal of Vascular & Endovascular Surgery.

[4] Bethesda M.D. National Center for Biotechnology Information, National Library of Medicine. http://www.ncbi.nlm.nih.gov/SNP/.

[5] Amos C.I. (2007). Successful design and conduct of genome-wide association studies. Human Molecular Genetics.

[6] Wellcome Trust Case Control Consortium (2007). Genome-wide association study of 14,000 cases of seven common diseases and 3,000 shared controls. Nature.

[7] Helgadottir A., Thorleifsson G., Magnusson K.P., Gretarsdottir S., Steinthorsdottir V., Manolescu A. (2008). The same sequence variant on 9p21 associates with myocardial infarction, abdominal aortic aneurysm and intracranial aneurysm. Nature Genetics.

